# The Role of Axial Length and Intraocular Pressure in the Follow-Up of Growing Children with Primary Congenital Glaucoma

**DOI:** 10.3390/jcm14072152

**Published:** 2025-03-21

**Authors:** Rosa Longo, Elia Franzolin, Elena Gusson, Francesca Chemello, Silvia Panunzi, Giorgio Marchini

**Affiliations:** 1 Ophthalmic Unit, Department of Surgical Odontostomatological Maternal and Child Sciences, Integrated University Hospital of Verona, 37134 Verona, Italy; 2 Unit of Epidemiology and Medical Statistics, Department of Diagnostic and Public Health, University of Verona, 37129 Verona, Italy

**Keywords:** congenital glaucoma, infantile glaucoma, primary congenital glaucoma, glaucoma surgery, axial length, intraocular pressure, eye anatomy, visual acuity

## Abstract

**Purpose:** The aim of this study was to investigate the factors that affect ocular growth in children with primary congenital glaucoma (PCG) who have undergone surgical treatment. We designed an analysis like that proposed by Al-Obaida et al. to assess whether similar results could be obtained in the Caucasian population. **Methods:** Data on axial length (AxL), intraocular pressure (IOP), and corneal diameter of PCG patients were recorded and analyzed. **Results:** Patients’ age was significantly influencing the increase in AxL. Also, levels of IOP, number of glaucoma medications, and number of surgeries along the follow-up period were found to significantly influence AxL progression. **Conclusions:** This study confirms that in PCG patients, there is a curvilinear relationship between AxL and age, which is strongly influenced by IOP, gender, the number of surgical interventions, and the number of anti-glaucoma medications. These data seem to suggest that maintaining IOP values closer to normal ones for age is crucial in the first up to three years of life to avoid permanent ocular anatomic and functional damage caused by elevated intraocular pressure.

## 1. Introduction

Primary congenital glaucoma (PCG) is a rare disease caused by the dysgenesis of the trabecular meshwork and sclero-corneal angle, which leads to an increase in intraocular pressure (IOP) due to the obstacle to the normal outflow pathways of the aqueous humor [1,2].

In infants and young children, the sclera is thinner and less rigid than in adults.

This is the reason why a prolonged increase in IOP causes distension of the eyeball (buphthalmos), which leads to an increase in axial length.

This phenomenon is exclusive to childhood since, in adults, the sclera is stiffer and has greater resistance to distension [3]. Secondary alterations to this abnormal growth are corneal edema and opacities, Haab’s striae, and progressive optic nerve cupping [4].

PCG affects about 1/100,000 live births in Western countries, with an incidence approximately four times higher in the Middle East [2,5,6,7,8,9].

Axial length (AxL) is the main objective measurement used in the follow-up of infants since it is not possible to perform visual field testing in infants or very young children [10,11]. AxL values far from the normative ones or a rapid increase in AxL is, therefore, an indication of poor control of the disease.

That being said, it is often challenging to recognize when variations in AxL are due to physiological growth and when they are pathological [12].

Several studies on PCG patients have been conducted that evaluate AxL increase in relation to age [12,13]. Growth of AxL occurs in three stages throughout life. The first phase goes from birth to 2 years, during which there is rapid ocular growth of about 4 mm in the first six months of life and a further 2 mm until the year of age. The second phase lasts from 2 to 5 years, the third from 6 to 13 years approximately. In these two phases, an additional 2 mm (1 mm per phase) can be acquired [14].

A recent study has proposed a predictive model of AxL growth in patients affected by PCG that considers age, IOP values, and the patient’s gender [12]. One of the limitations of that study was the ethnicity of the patients, which did not include Caucasians.

The aim of this study is to validate the proposed model on a cohort of Caucasian patients and to create growth curves investigating factors that affect ocular growth in PCG.

## 2. Methods

Patients with PCG undergoing surgery at the Glaucoma Unit of the University Hospital of Verona between December 1996 and June 2017 were included in this retrospective study. The study protocol was previously approved by the local Ethics Committee. Informed consent to the surgical interventions has been obtained from a parent and/or legal guardian. All data were collected anonymously and following the ethical standards of the Declaration of Helsinki.

PCG was diagnosed based on clinical findings, following the criteria proposed by the Childhood Glaucoma Research Network (CGRN) [1,15].

All patients having retinal (i.e., Retinopathy of Prematurity) or inflammatory ocular comorbidities or systemic syndromes (i.e., Sturge–Weber Syndrome) were excluded. Glaucoma associated with acquired ocular anomalies or non-acquired ocular anomalies (Axenfeld–Rieger anomaly, Aniridia, Peters anomaly) were excluded. Patients for whom we did not have all the data necessary for the study or patients with secondary glaucoma were also excluded.

Both eyes of patients with bilateral pathology and only the pathological eye of patients with unilateral pathology were analyzed.

Intraocular pressure (IOP), horizontal corneal diameter (HCD), corneal transparency, axial length (AL), ON damage (defined as cup-to-disk ratio >0.5), number of administered drugs, and number of surgical procedures were analyzed.

Children <5 years of age were examined under inhalation anesthesia before intubation or under sedation during a follow-up visit (using inhaled Sevorane 250 mL, which has no significant effect on IOP measurements) [16,17]; those >5 years of age were examined without inhalation anesthesia.

The first performed measurement was the IOP, with a portable applanation tonometer (Perkins tonometer).

AxL measurements were taken by only one experienced ophthalmologist (GM) to avoid bias and ensure correct recording of data, utilizing a 10 MHz A-scan machine (Compact Touch ^®^, Quantel Medical, Cournon-d’Auvergne, France) using contact biometry after application of a topical anesthetic agent (Lidocaine Hydrochloride 40 mg/mL eye drops). In each patient, the machine recorded at least ten accurate AL measurements and gave an average reading of all measurements (mean of at least 10 measurements ± standard deviation) and considering the greater anterior chamber depth.

Horizontal corneal diameter (white-to-white) was measured using a surgical caliper.

The number of anti-glaucoma medications and the number and type of surgeries were also reported at each follow-up.

## 3. Statistical Analysis

Baseline characteristics were described as reporting categorical variables as frequencies and percentages and continuous data with their mean and standard deviation or median and interquartile range.

AxL progression in time was analyzed for each of the patient’s eyes at baseline (patient’s first access in the clinic) and at 1, 3, and 5 years after the first surgery.

An exploratory analysis was first conducted to visualize measures of AxL (mm) with respect to the patient’s age (months) and for two IOP cut-offs, separately (IOP ≤ 21 mmHg and IOP > 21 mmHg) on any visit; this cut-off is typically used to classify abnormally high IOP in glaucoma studies involving children [1,3,10,18,19,20,21,22,23,24,25,26,27,28,29].

Nonparametric curves were then fitted to the data to estimate the relationship of AxL and age for the two groups.

Parametric linear mixed model (LMM) regression analysis was then used to model the effect of age and level of IOP on AxL for all included eyes, using age, gender, IOP, number of medications, and number of surgeries as explanatory variables and patient and eyes as random intercepts to account for multiple observations. Five LMMs were developed, from the null to a complete model with all variables included. The model with the lowest 2 Log Likelihood and AIC value was considered to have the best fit.

Models’ formulation starts with Model 1, which includes a quadratic spline to model the relationship between the outcome (AxL) and age (months) of the patients to better capture the time-changing shape of the axial length growth curve. Model 2 also includes IOP and gender. Model 3 further includes bilaterality (yes/no), Model 4 includes the number of anti-glaucoma medications (AGM) assumed, and Model 5 includes the number of surgeries during follow-up. Model 6 was the complete model in which the interaction of the level of IOP during visit time and the patient’s gender was also investigated. The chosen model, those having better selection criteria, was Model 5. From Model 5, all the following prediction plots were derived. Table 1 shows the study models.

To assess the goodness of fit of the models, we used the Akaike Information Criterion (AIC) and the Bayesian Information Criterion (BIC). AIC and BIC are indicators that evaluate how well a model fits the data while penalizing model complexity to prevent overfitting. Both criteria were applied, and the chosen model was the one (Model 5) having the lowest AIC and BIC.

Statistical analysis was carried out with R software version 4.0.3. A *p*-value <0.05 was considered statistically significant.

## 4. Results

The study sample included 101 eyes from 62 PCG patients. Table 2 shows baseline data for patients in our study population.

**Figure 1:**
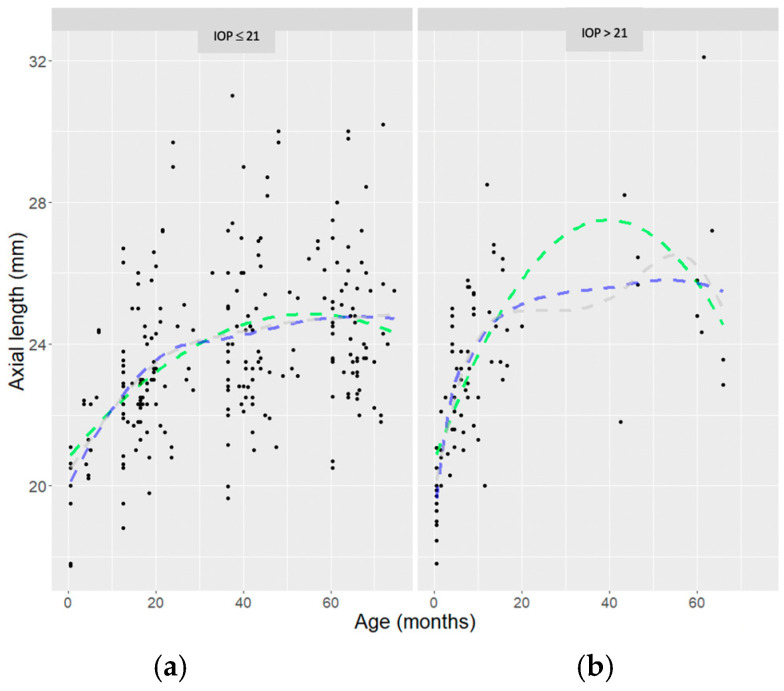
Lowess and quadratic functions fitting raw children’s data on axial length (AxL, mm) plotted by children’s age (months) and stratified by level of intraocular pressure (IOP): ≤ 21 mmHg (**a**) and >21 mmHg (**b**). (blue = lowess function; green= quadratic spline; gray = simple quadratic fit).

Half of the patients were males (51.6%), and half were females (48.4%). All patients underwent glaucoma surgery. The mean age at first surgery was 5.7 ± 4.8 months (mean ± SD). The median of observations under general anesthesia was 8.00 (Inter Quartile Range—IQR 6.00, 10.00). The median of the surgical procedures was 2.00 (IQR 1.00, 3.00). The mean patient’s age was 12.36 months. Patients’ follow-up time (range min–max) was a mean of 82.3 (54.8–114.4) months. A total of 26% of patients had at the time of surgery an ongoing therapy with Timolol eye drops or Latanoprost eye drops, awaiting surgery. Figure 1 shows the raw AxL data plotted against age, with a lowess function and quadratic curves fitting the data. There was a non-linear relation between AxL and the age (months) of the patients for both the IOP groups (IOP ≤ 21 or >21 mmHg) at any time point. Conversely, the curve for IOP > 21 mmHg was steeper at all the corresponding time points.

All the linear mixed models formulated for the analysis of eyes’ AxL during follow-up are shown in Table 2. Patients’ age (months) was, as expected, significantly influencing the increase in AxL. In addition, levels of IOP, number of glaucoma medications, and number of surgeries along the follow-up period were also found to significantly influence AxL progression. The final model (Model 5), with the lowest AIC values, comprises age, IOP, gender, bilaterality, AGM, and number of surgeries needed as independent variables.

**Figure 2:**
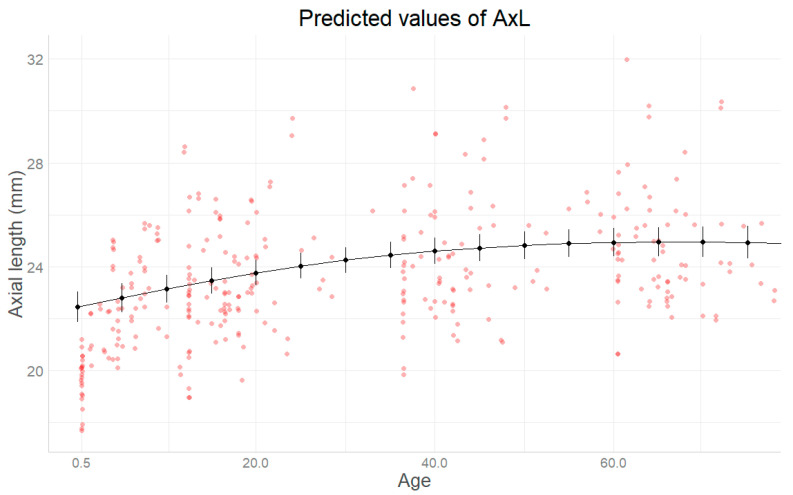
Predicted mean axial length (AxL) (with 95% confidence intervals, CIs) at different patients’ ages (months).

**Figure 3:**
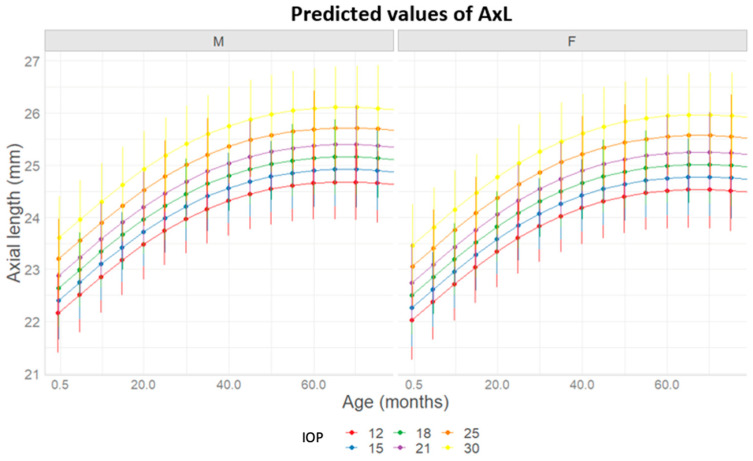
Gender-specific differences in axial length (AxL) increase, predicted for different predetermined levels of intraocular pressure (IOP).

**Figure 4:**
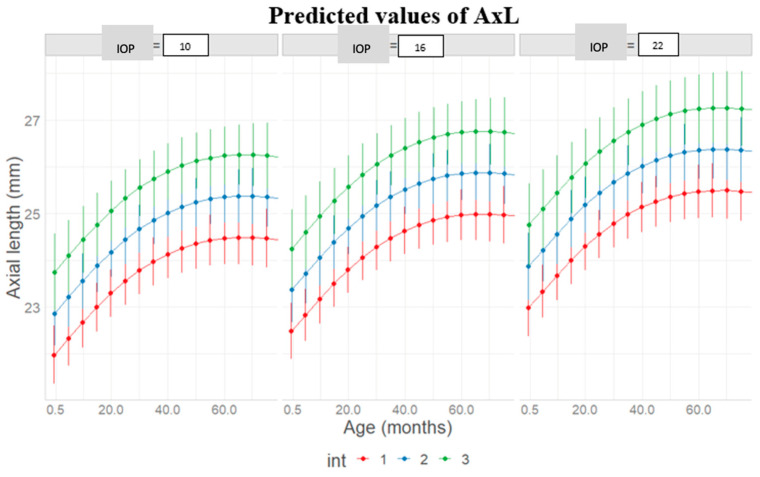
Predicted axial length (AxL) increase with respect to age (months), intraocular pressure (IOP) (mmHg) levels, and number of surgical interventions (int) during follow-up.

**Figure 5:**
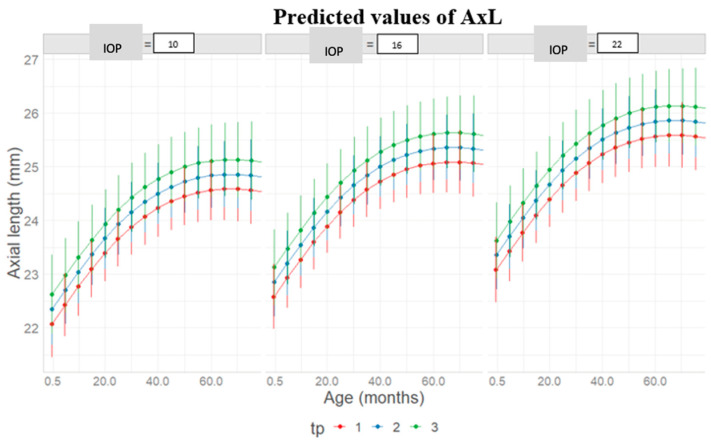
Predicted axial length increase with respect to age (months), intraocular pressure (IOP, mmHg) levels, and number of anti-glaucoma medications (tp) undertaken during follow-up.

The mean predicted curve derived from the final model is shown in Figure 2. AxL was not increasing differently between genders, with curves reaching their pick of increase at around 60 months for both (Figure 3). Also, AxL was shown to increase slightly in a steeper way when increasing the number of therapies or the number of surgeries during follow-up time (Figure 4 and Figure 5).

## 5. Discussion

AxL is currently the most reliable monitoring index for the progression of congenital glaucoma [30,31]. In fact, the corneal diameter stabilizes soon after surgery, and the assessment of optic nerve cupping is not always accurate due to the opacity of the dioptric media [22,24,28,32]. Moreover, optic nerve cupping is reversible in children due to the distensibility of the cribrous plate [21,33].

As already stated by previous studies, the growth in axial length does not have a linear relationship with time. In fact, the greatest curvilinear growth occurs in the first six months of life and up to one year of life. The growth rate then slows until it reaches a plateau around 3 years [34].

The objective of this study was to investigate the factors that affect ocular growth in children with PCG who have undergone surgical treatment. We designed an analysis similar to that proposed by Al-Obaida et al. [12] to assess whether similar results could be obtained in the Caucasian population. A five-level model was used to evaluate the influence of age, IOP, and the number of surgical interventions and anti-glaucoma medications.

All enrolled patients underwent surgery, while in the study by Al-Obaida et al., 41.1% of the total (163 eyes) had undergone surgery.

Similarly to Al-Obaida et al., we found a curvilinear relationship between AxL and age, which was strongly influenced by IOP, the number of surgical interventions, and the number of anti-glaucoma medications.

AxL reaches a plateau around 3 years of age, similar to children without pathology, potentially due to the decreased distensibility of the sclera after that age [32].

These data seem to suggest that maintaining the IOP at the lowest values is crucial in the first three years of life to limit the progression of AxL elongation and, consequently, myopia and its pathological complications.

Our data indicate that the arrest of ocular growth in PCG patients begins between 45 and 60 months of age, confirming previous findings proposed by Fledelius, Kiefer, and Law [30,35,36].

Furthermore, AxL is consistently greater in males than females, revealing a greater predisposition to the growth of AxL in males, as proposed in other previous studies [10,34]. Males, in fact, have AxL values significantly higher than those of females at all ages, Figure 2. This suggests the possibility that females have greater resistance to the increase in AxL.

The increased AxL is then an indicator of poor control of the pathology; patients with higher AxL are those who have required a greater number of surgical interventions and antiglaucoma medications; Figure 4 and Figure 5.

As a result, AxL at birth influences future growth. Even if stabilized at lower IOP, the eyes continue their growth, resulting in higher-than-normal AxL values and subsequent moderate to severe axial myopia [10,34].

Another aspect analyzed is the role of IOP in relation to AxL and age. From Figure 1, we can see how the steepness of the growth curve in the first year of life is evident for IOP values higher than 21 mmHg. This clarifies that the role of IOP control in the management of the pathology is fundamental and supports what has been highlighted by other studies regarding the early timing of surgery for congenital glaucoma [11,37].

Eyes with lower IOP values (12–15 mmHg) exhibited a slowdown in upper AxL growth, similar to eyes unaffected by the disease. This suggests that maintaining IOP around the normal values of 10–12 mmHg is necessary to prevent excessive AxL growth. Poorly controlled pathology leads to AxL elongation and the need for reoperations [3,22,25,27,28,37,38].

The strengths of this study include the large sample size, despite the relative rarity of the disease in Western countries, and the long-term follow-up, with an average of about 7 years. It would be useful, with the aim of encouraging data sharing and increasing knowledge on the topic, to establish a shared web tool for the international collection of data on the management of congenital glaucoma, as previously suggested.

The main limitation of the study is its retrospective design. Another limitation is the absence of a control group. We did not include a group of healthy patients as it is difficult to find neonates or very small patients to undergo AxL measurement under general anesthesia, as this raises ethical questions. For this reason, we referred to studies on healthy subjects in the scientific literature [32,34,35].

In conclusion, this study confirms that in PCG patients, AxL is primarily influenced by age and IOP. Additionally, the number of surgical interventions and anti-glaucoma medications correlate with globe enlargement and are partly attributed to poor IOP control. These findings highlight the importance of referring these patients as soon as possible to highly specialized centers, as the initial therapeutic approach is crucial for avoiding the disease’s progression.

## Figures and Tables

**Table 1:** LMM estimated effects and confidence intervals [95% CI], models studying the association between axial length growth and age of children (ns, natural spline). Models 1 to 5 describe results sequentially adjusting for further covariates: intraocular pressure (IOP), gender, bilaterality, number of therapies (tp), number of interventions. Model 6 investigates the interaction between level of IOP during follow-up and patient’s gender. Information criteria (Akaike Information Criteria, AIC, and Bayesian Information Criteria, BIC) were also reported. “ns(age, 2)1” and “ns(age, 2)2” refer to the two terms of the natural spline for age, applied to two different groups, separated by IOP levels. The ’1’ refers to the first group (IOP ≤ 21 mmHg), while the ’2’ refers to the second group (IOP > 21 mmHg). * statistically significant.

	Model 1	Model 2	Model 3	Model 4	Model 5	Model 6
(Intercept)	21.89 [21.40; 22.38] *	21.25 [20.45; 22.06] *	21.68 [20.60; 22.76] *	21.48 [20.41; 22.55] *	20.66 [19.59; 21.74] *	20.54 [19.43; 21.65] *
ns(age, 2)1	5.43 [4.57; 6.30] *	6.09 [5.13; 7.05] *	6.05 [5.09; 7.00] *	6.00 [5.04; 6.97] *	4.39 [3.40; 5.38] *	4.40 [3.41; 5.39] *
ns(age, 2)2	0.62 [−0.58; 1.82]	0.38 [−0.82; 1.58]	0.38 [−0.82; 1.58]	0.31 [−0.87; 1.49]	0.81 [−0.28; 1.90]	0.79 [−0.30; 1.89]
IOP		0.03 [0.01; 0.05] *	0.03 [0.01; 0.05] *	0.03 [0.02; 0.05] *	0.08 [0.06; 0.10] *	0.09 [0.06; 0.11] *
Gender (F)		−0.21 [−1.10; 0.68]	−0.23 [−1.12; 0.66]	−0.18 [−1.07; 0.70]	−0.14 [−1.03; 0.75]	0.09 [−0.93; 1.11]
Bilateral			−0.58 [−1.54; 0.39]	−0.65 [−1.60; 0.31]	−0.80 [−1.75; 0.15]	−0.80 [−1.76; 0.15]
Therapies				0.37 [0.21; 0.52] *	0.27 [0.13; 0.41] *	0.28 [0.14; 0.42] *
Interventions					0.88 [0.67; 1.10] *	0.88 [0.67; 1.10] *
IOP:Gender (F)						−0.01 [−0.04; 0.01]
IOP:N.Interventions	1130.14	1132.87	1133.09	1098.53	1047.96	1055.70
AIC	1172.29	1182.61	1186.61	1155.52	1108.70	1120.18
BIC	−554.07	−553.43	−552.54	−534.27	−507.98	−510.85
Log Likelihood	344	344	344	337	337	337
Num. obs.	101	101	101	101	101	101
Num. groups: eye	62	62	62	62	62	62
Num. groups: patient	21.89 [21.40; 22.38] *	21.25 [20.45; 22.06] *	21.68 [20.60; 22.76] *	21.48 [20.41; 22.55] *	20.66 [19.59; 21.74] *	20.54 [19.43; 21.65] *

**Table 2:** Baseline characteristics of study population (101 eyes of 62 children) at the first visit. SD, standard deviation; IOP, intraocular pressure.

Parameter	Value
Age (months), mean ± SD	12.36 (±37.44)
Gender (boys: girls), number of eyes (%)	32:30; 51.6:48.4
Number of anti-glaucoma medications, mean ± SD	0.26 (±0.64)
IOP (mmHg), mean ± SD	24.11 (4.28)
Axial length (mm), mean ± SD	22.45 (±2.37)
Previous glaucoma surgery, number of eyes (%)	100%

## Data Availability

Data are available upon reasonable request.
